# Reliability of a Novel CBCT-Based 3D Classification System for Maxillary Canine Impactions in Orthodontics: The KPG Index

**DOI:** 10.1155/2013/921234

**Published:** 2013-10-09

**Authors:** Domenico Dalessandri, Marco Migliorati, Rachele Rubiano, Luca Visconti, Luca Contardo, Roberto Di Lenarda, Conchita Martin

**Affiliations:** ^1^Department of Orthodontics, School of Dentistry, University of Brescia, Piazzale Spedali Civili 1, 25123 Brescia, Italy; ^2^Department of Medical, Surgical and Health Sciences, School of Dentistry, University of Trieste, Piazza Ospitale 1, 34129 Trieste, Italy; ^3^Department of Orthodontics, School of Dentistry, University of Genova, Viale Benedetto XV 6, 16132 Genova, Italy; ^4^Department of Orthodontics and Gnathology-Masticatory Function, Dental School, University of Turin, Via Nizza 230, 10100 Turin, Italy; ^5^Department of Stomatology IV, School of Dentistry, Complutense University of Madrid, Plaza Ramón y Cajal s/n, 28040 Madrid, Spain

## Abstract

The aim of this study was to evaluate both intra- and interoperator reliability of a radiological three-dimensional classification system (KPG index) for the assessment of degree of difficulty for orthodontic treatment of maxillary canine impactions. Cone beam computed tomography (CBCT) scans of fifty impacted canines, obtained using three different scanners (NewTom, Kodak, and Planmeca), were classified using the KPG index by three independent orthodontists. Measurements were repeated one month later. Based on these two sessions, several recommendations on KPG Index scoring were elaborated. After a joint calibration session, these recommendations were explained to nine orthodontists and the two measurement sessions were repeated. There was a moderate intrarater agreement in the precalibration measurement sessions. After the calibration session, both intra- and interrater agreement were almost perfect. Indexes assessed with Kodak Dental Imaging 3D module software showed a better reliability in *z*-axis values, whereas indexes assessed with Planmeca Romexis software showed a better reliability in *x*- and *y*-axis values. No differences were found between the CBCT scanners used. Taken together, these findings indicate that the application of the instructions elaborated during this study improved KPG index reliability, which was nevertheless variously influenced by the use of different software for images evaluation.

## 1. Introduction

Since a long time, impacted maxillary canines treatment has been an interesting challenge, both from the diagnostic and the therapeutic point of view, for every orthodontist [1–3]. Traditional methods of 2D radiological imaging, such as orthopantomogram (OPG), cephalometric radiography, and intraoral occlusal or periapical X-rays, were routinely used for diagnostic purposes [4–6]. 3D computed tomography was usually requested only for evaluating or detecting dental root reabsorptions, or in patients with particular pathologies [7], because of the high X-ray dose administered to the patient by these traditional multislices computed tomography (MSCT) scanners. 

Recently CBCT, a new CT technology with a reduced X-ray emission, was invented and, during the last decade, there was a rapid increase of clinical applications of these scanners [8]. CBCT reliability was demonstrated to be accurate enough for maxillofacial [9–11], orthodontic [12–14], and dental implantology purposes [15]. CBCT was initially used as a substitute of MSCT in special needs patients [16–18] and in dental impactions [19] or supernumerary teeth [20] diagnosis, but currently its clinical application field is rapidly widening.

**Figure 1:**
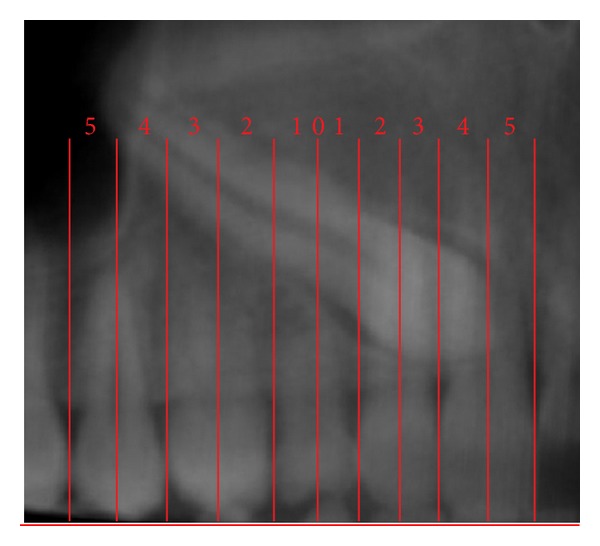
Mesiodistal position (*x*) for both cusp and root tips; Panorex view. In this example *C*_*x*_ = 5 and *R*_*x*_ = 5.

**Figure 2:**
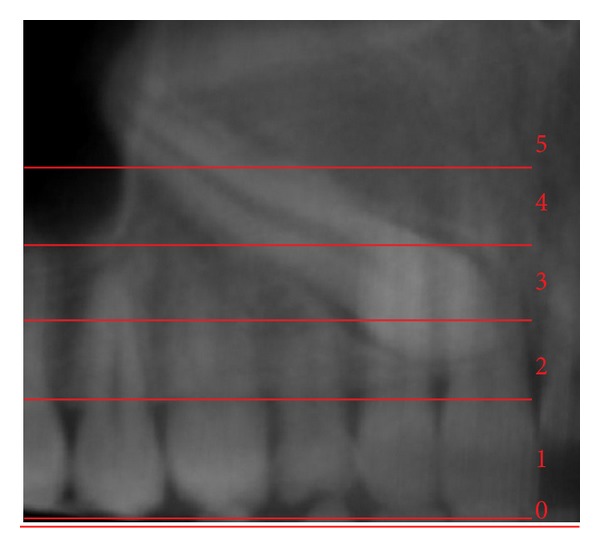
Vertical position (*y*) for cusp tip; Panorex view. In this example *C*_*y*_ = 2.

**Figure 3:**
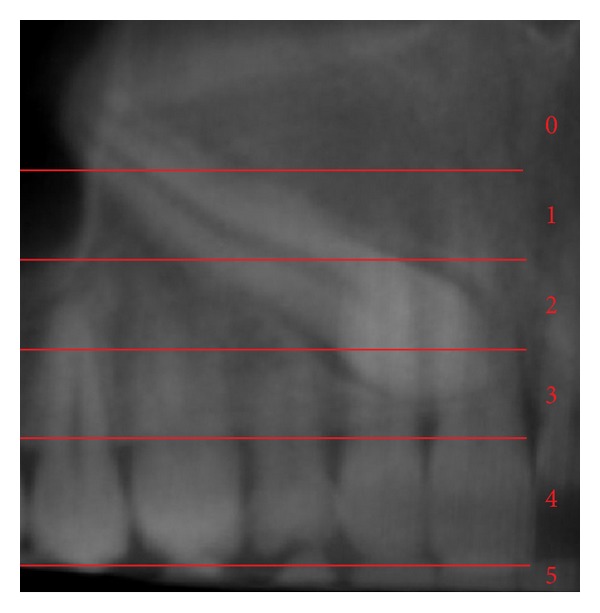
Vertical position (*y*) for root tip; Panorex view. In this example *R*_*y*_ = 0.

**Figure 4:**
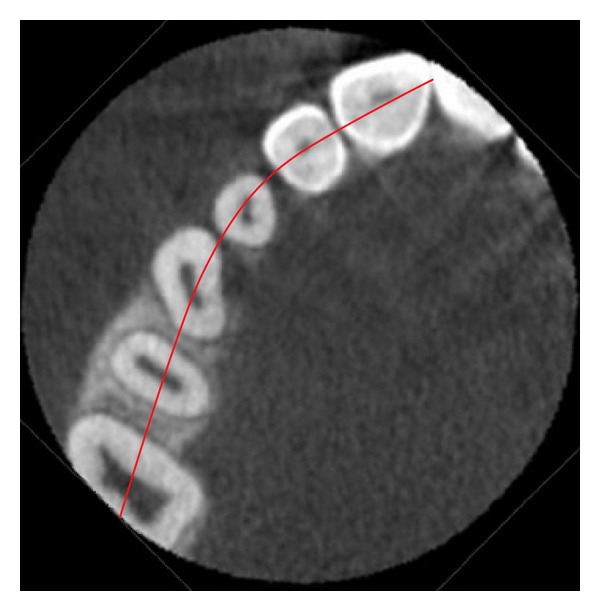
Occlusal reference arch (*z*); axial view. In this example *C*_*z*_ = 0 and *R*_*z*_ = 0; therefore the KPG index value is 12—moderate difficulty (5 + 5 + 2 + 0 + 0 + 0 = 12).

In 2009, a novel method of analyzing maxillary canine impactions was proposed, the KPG index [21]. This index classifies the canine's position, based on their distance from the norm, giving a number on a 0–5 scale to both cusp and root tip along *x*, *y*, and *z* planes (Figures 1, 2, 3, and 4). The sum of these six scores would assess the anticipated difficulty of treatment, classified as easy (0–9), moderate (10–14), difficult (15–19), and extremely difficult (20 and above). The authors of this index used the images of 42 impacted canines obtained with the Sirona Galileos CBCT scanner and they analyzed them with the Galaxis software.

The ability of this index to provide an estimate of the time necessary to treat an impacted canine was recently investigated [22], but the ease of use and the repeatability of this index quantifications are still unknown.

Thus, the aim of this study was to assess both inter- and intrarater reliability of the measurements of KPG index taken on images obtained with different CBCT scanners and analyzed with different 3D visualization software.

## 2. Materials and Methods

CBCT exams of 50 impacted canines were collected from three different radiological centers. 12 canines were studied with a NewTom 3G scanner set at 0.3 mm voxel and 15 × 15 cm Field of View (FOV) sizes, with a slice interval of 1 mm; 13 canines with a Kodak 9500 scanner set at 0.3 mm voxel and 15 × 9 cm FOV sizes, with a slice interval of 1 mm; and 25 canines with a Planmeca Promax Mid scanner set at 0.2 mm voxel and 16 × 9 cm FOV sizes, with a slice interval of 1 mm. 

Digital Imaging and Communications in Medicine (DICOM) files obtained with the first two scanners were visualized with the Kodak Dental Imaging 3D module software, whereas Planmeca Promax scanner images were visualized with the Planmeca Romexis software. All the images were visualized on a 16 : 9 27′′ Light Emitting Diodes (LED) backlighting monitor display (iMac, Apple, Cupertino, CA, USA) with a 2560 × 1440 pixel screen resolution.

Three orthodontists, after reading the manuscript where the KPG index was proposed for the first time, were asked to independently assess these 50 canines using this index (*t*_0_). Measurement sessions on the same canines were repeated one month later (*t*_1_). Based on this first experience, they found an agreement about few guidelines in applying this index. A joint calibration session, providing the same guidelines, was organized with nine orthodontists one month after the *t*_1_ session and the two measurement sessions were repeated, again with a one month interval between the first (*t*_2_) and the second (*t*_3_) ones.

### 2.1. Statistical Analysis

The reliability of the KPG index was tested verifying agreement between two different times for each rater (intraobserver agreement) and agreement among different raters (interobserver agreement). 

Because KPG index is an ordinal variable, Cohen's kappa coefficient was quantified to assess intraobserver agreement and the Kendall coefficient of concordance (Kendall's W) was quantified to assess interobserver agreement.

Both coefficients range from 0 to 1, with higher values indicating a stronger relationship: values ≤0.01 indicate poor agreement, values between 0.01 and 0.20 slight agreement, between 0.21 and 0.40 fair agreement, between 0.41 and 0.60 moderate agreement, between 0.61 and 0.80 substantial agreement, between 0.81 and 0.99 almost perfect agreement, and 1 perfect agreement. 

As additional information, the percentage of agreement and the percentage of disagreement were calculated. Percentage of disagreement was divided into cases where the disagreement was in one category (one stage apart) or in more than one category (two stages apart).

All the measurements were statistically analyzed using SPSS Statistics version 19 (SPSS Inc., Chicago, IL) software.

## 3. Results

### 3.1. First Session Results

Data were analyzed only considering together all results obtained with different software and scanners, without investigating differences eventually present pertaining each singular axial value that contributes to the definition of the final KPG index total value. 

Intra-rater agreement between *t*_0_ and *t*_1_ showed a kappa coefficient of 0.417 and a percent agreement, respectively, of 48% for rater Domenico Dalessandri, of 0.465 and 52% for rater Marco Migliorati, and of 0.490 and 54% for rater Rachele Rubiano, statistically indicating moderate agreement. One stage apart disagreement was 52% for rater Domenico Dalessandri, 46% for rater Marco Migliorati, and 46% for rater Rachele Rubiano. 

Kendall's *W* values were 0.940 at *t*_0_ and 0.899 at *t*_1_, thus demonstrating a strong interrater statistical agreement. 

### 3.2. Operative Recommendations Proposal

At the end of *t*_1_, the three orthodontists expressed their doubts and difficulty using the KPG, which were summarized in the following questions.

- Do we have to maintain the spatial orientation of the acquired volume or do we have to reorientate it accordingly to specific reference planes?
- Which are the decisional criteria to assign the lower or the higher score if the cusp or root tip falls on the junction of two sections, when assessing *x*- and *y*-axis?
- Regarding *z* plane, which is the definition of “occlusal reference arch”?
- How must the correct axial-plane be located with reference to this arch?
- Should distances along the *z* plane be measured perpendicularly to the occlusal arch, as stated in the KPG article, or from the cusp/root tip to the proper canine cusp tip location along the occlusal arch, as shown in Figure  6 of [21]?
- Should the proper canine cusp/root tip location considered be in the center of the alveolar bridge, as it seems to look at Figure 4 of the KPG manuscript?

After a discussion session, the following recommendations were defined.

- In case of evident wrong patient positioning during the CBCT exam, it is appropriate to reorientate the volume maintaining the maxillary plane parallel to the axial *z* plane and eliminating rotations around *y*-axis (sagittal median plane).
- In case of doubt in scoring a parameter, take into account teeth general position and characteristics. For example, reduced canine root length or augmented premolar root length could alter *y* scoring of canine root tip; it is important in this case to evaluate if angulation of the canine is really augmented or not, and then choose the lower score if canine long axis is quite vertical. On the other hand, highly malpositioned laterals or premolars could alter evaluations regarding *x*-axis. In case of doubts regarding several of the scores, it is preferable to choose alternately the higher and the lower of the two considered values for each score.
- “Occlusal reference arch” is the curved line, drawn on an axial plane that passes through the centers of the clinical crowns of all the teeth, when they are correctly aligned. The correct axial plane for individuating this arch is the one going through the necks of teeth.
- Distances along the *z* plane must be measured perpendicularly to the occlusal reference arch. A measure taken from the cusp/root tip to the proper canine cusp tip location is influenced also from their mesiodistal position that is still considered in measures along the *x* plane: this sum of effects on measurements must be avoided to prevent scoring alterations.
- The proper canine cusp/root tip location is considered to be in the center of the alveolar bridge because this is the ideal position for cusp tip eruption. Surely, when the canine is fully erupted, the final ideal position of both cusp and root tips is not the center of the alveolar bridge, but is more vestibular for the cusp tip and more palatal for the root tip, depending on the final canine torque value.

### 3.3. Second Session Results

Table 1 shows kappa coefficients between *t*_2_ and *t*_3_, considering each rater individually. They ranged from 0.676 to 0.930, statistically indicating substantial or in some cases almost perfect intra-rater agreement. Overall percent agreement was 82.4%, one stage and two stages apart disagreement were 16.7% and 0.9%, respectively (Table 2). 

Kendall's *W* values were 0.970 at *t*_2_ and 0.992 at *t*_3_, thus demonstrating an almost perfect interrater statistical agreement. The percent agreement values were 81.1% at *t*_2_ and 95.3% at *t*_3_; one stage apart disagreement values were 18.2% and 4.7%, respectively; two stage apart disagreement values were 0.7% and 0.0%, respectively (Table 3).

Data were subsequently analyzed separating KPG index in its six components (cusp on *x*, *y*, and *z* planes—*C*_*x*_, *C*_*y*_, and *C*_*z*_; root on *x*, *y*, and *z* planes—*R*_*x*_, *R*_*y*_, and *R*_*z*_) and comparing results obtained using different software and scanners.

*K* values of images visualized with the Kodak Dental Imaging 3D module software and obtained with NewTom 3G and Kodak 9500 scanners, both set at 0.3 mm voxel size with a slice interval of 1 mm, were substantially equivalent, considering each rater separately (Table 4). Kendall's *W* values were 0.971 and 0.992 for NewTom 3G and 0.934 and 0.969 for Kodak 9500, respectively, at *t*_2_ and *t*_3_.

*K* values of images visualized with the Kodak Dental Imaging 3D module software were higher when considering *C*_*z*_ and *R*_*z*_, and were lower when considering *C*_*x*_, *C*_*y*_, *R*_*x*_, and *R*_*y*_, compared with values of images visualized with the Planmeca Romexis software (Table 5). The same tendency was found comparing Kendall's *W* values (Table 6).

## 4. Discussion

Orthodontic treatment of impacted canines requires accurate localization to surgically expose and retrieve each tooth most efficiently, individualizing clinical approach and mechanics [23]. CBCT, maintaining the ability to eliminate the overlapping of contiguous structures, to precisely detect root reabsorption of adjacent teeth, and reducing the radiation dose if compared with MSCT [24], is currently suggested to be the most suitable radiological exam when treating impacted canine patients [25, 26]. 

The KPG index was proposed as a simple method to locate and assign a difficulty score to impacted maxillary canines using CBCT. If this ability will be confirmed by prospective studies, KPG index could become a very useful tool for every orthodontist in estimating individually treatment time necessary to bring the canine to its proper position. 

The first aim of our study was to assess KPG index reproducibility, because firstly, we think that it is of crucial importance to establish if this index is really easy to score and if it gives repeatable results when the same patient is assessed by different operators or by the same operator in different sessions. In fact, before evaluating the validity of a new clinical index, it is important to test its reproducibility; for example, the cervical vertebral maturation (CVM) method, an index used to assess patient maturational age that was recently proposed in an improved version [27, 28] and then widely applied in evaluating clinical effect of orthopedic treatment timing in orthodontics, is now under revision by recent studies [29–31]. Initial results of our study showed a moderate inter-rater agreement, demonstrating that individual anatomical situations could be differently interpreted and assessed by different operators, when measuring references are not exactly and widely explained. However, after drawing further clarifications from a calibration session, the inter-rater agreement increased to almost perfect, thus demonstrating the reliability of this index. 

Our second aim was to evaluate visualization software influences on KPG scores. In fact, the inventors of this index always used the Galaxis software, which is not used by all clinicians; therefore, it is important to obtain a high reproducibility regardless of the software used. We found differences between the two softwares that we used, probably because of their specific features. Indexes assessed with Kodak Dental Imaging 3D module software showed a better reliability in *z*-axis values than in *x*- and *y*-axis values. This could be because of two reasons: first, the possibility to set, on an axial plane, the point from which the measurement begins and then scroll through the other sections until reaching the end measurement point, making it easy to correctly register *z*-axis measurement; second, the limited thickness of slices analyzed on the Panorex view, which complicates evaluation on *x*- and *y*-axis if impacted tooth is far from the curve where other teeth lie. On the other hand, indexes assessed with Planmeca Romexis software showed a better reliability in *x*- and *y*-axis values than in *z*-axis values. Again this could be because of two reasons: first, the need to manually fix on the screen, on an axial plane, the point from which the measurement begins and then scroll through the other sections until reaching the end measurement point; however, this facilitates making mistakes when registering *z*-axis measurement; second, the possibility to set an OPG-like thickness of slices analyzed on the Panorex view, obtaining an image with all teeth easily visible thus facilitating the evaluation on *x*- and *y*-axis even if impacted tooth is far from the dental arch. 

The third aim of this study was to investigate if the CBCT scanner employed to obtain 3D radiological images could influence KPG index score. In fact, several studies [32, 33] demonstrated that different CBCT scanners could have different measurement reliability and accuracy depending not only on voxel size but also on technical setting (kV, mA, exposure time, and focal spot dimensions) and sensor technology (flat panel, brilliance intensifier). Therefore, we decided to compare two different scanners, a NewTom 3G (equipped with an image intensifier sensor, similar to the Sirona Galileos utilized in the first KPG study) and a Kodak 9500 (equipped with a flat panel sensor), both set with a voxel dimension of 0.3 mm and a slice interval of 1 mm. We took this decision because currently there are many different CBCT scanners available on the market; therefore, it is difficult to standardize a protocol based on a particular scanner: we think that it is more useful to define acquisition parameters settings that could be used with all different scanners. Ideally, the voxel size should be smaller than the actual spatial resolution of the dataset, ensuring that the voxel size will not become the bottleneck when determining the spatial resolution. On the other hand, there is a limit in reducing voxel size when reconstructing datasets as a consequence of the file size and excessive increase of reconstruction time. Using this voxel dimension, which seemed to us to be a good compromise between image quality and file size, we found no differences between the two CBCT scanners used in this study, when images are analyzed using the same software. This could be because of the fact that (i) the canine is a high contrast structure, the boundary of which is easily delimited inside a less radiopaque structure such as the cancellous bone, thus allowing a good precision in measurements along the *z*-axis and (ii) that this submillimetric image definition is enough to allow a correct teeth visualization on OPG-like view, when scoring the KPG index along the *x*- and *y*-axis.

## 5. Conclusions

Our results demonstrate the following.

- KPG index intra- and inter-rater reliability could be unsatisfactory after only reading the manuscript in which it was proposed for the first time.
- With further detailed practical instructions, intra- and inter-rater reliability could rise to an almost perfect agreement level.
- Software used to assess impacted canines with this index must allow to obtain an OPG-like image for evaluating *x*- and *y*-axis scores and to digitally point the starting and the ending measurement points on axial slices for evaluating *z*-axis score.
- The KPG index reproducibility is not influenced by the CBCT scanner used, if voxel size and slice interval are equal.

## Figures and Tables

**Table 1:** Kappa coefficients for intrarater agreement between *t*_2_ and *t*_3_.

Observer	Kappa coefficient	Standard error
1	0.838	0.057
2	0.788	0.062
3	0.930	0.039
4	0.701	0.072
5	0.676	0.071
6	0.817	0.059
7	0.772	0.065
8	0.743	0.067
9	0.884	0.049

**Table 2:** Intrarater agreement and disagreement between *t*_2_ and *t*_3_.

KPG	Agreement percentage
Complete agreement	82.4% (371/450)
One stage apart	16.7% (75/450)
Two stages apart	0.9% (4/450)
Three stages apart	0% (0/450)

**Table 3:** Interrater agreement and disagreement at *t*_2_ and *t*_3_.

KPG	*t*_2_	*t*_3_
Complete agreement	81.1% (365/450)	95.3% (429/450)
One stage apart	18.2% (82/450)	4.7% (21/450)
Two stages apart	0.7% (3/450)	0% (0/450)
Three stages apart	0% (0/450)	0% (0/450)

**Table 4:** Kappa coefficients for intrarater agreement between *t*_2_ and *t*_3_, clustered by scanner type.

Observer	NewTom 3G		Kodak 9500	
	Kappa coefficient	Standard error	Kappa coefficient	Standard error
1	0.854	0.080	0.816	0.084
2	0.726	0.080	0.763	0.036
3	1	0.000	0.926	0.050
4	0.764	0.064	0.709	0.088
5	0.730	0.080	0.746	0.082
6	0.833	0.046	0.816	0.074
7	0.765	0.063	0.745	0.084
8	0.729	0.079	0.718	0.073
9	0.632	0.046	0.654	0.068

**Table 5:** Kappa coefficients for intrarater agreement between *t*_2_ and *t*_3_, clustered by software and KPG single component.

Observer	*C*_*x*_				*C*_*y*_				*C*_*z*_			
	Kodak		Planmeca		Kodak		Planmeca		Kodak		Planmeca	
	k*	SE**	k*	SE**	k*	SE**	k*	SE**	k*	SE**	k*	SE**
1	0.808	0.078	0.827	0.069	0.795	0.073	0.926	0.050	0.855	0.089	0.787	0.076
2	0.726	0.086	0.862	0.064	0.715	0.098	0.799	0.097	0.691	0.109	0.576	0.094
3	0.806	0.077	0.931	0.047	0.861	0.074	0.963	0.036	0.840	0.074	0.721	0.072
4	0.747	0.071	0.827	0.069	0.650	0.114	0.926	0.050	0.755	0.089	0.652	0.079
5	0.805	0.078	0.862	0.064	0.632	0.108	0.633	0.095	0.652	0.112	0.508	0.093
6	0.730	0.086	0.931	0.047	0.734	0.096	0.963	0.036	0.840	0.074	0.749	0.083
7	0.806	0.079	0.827	0.069	0.707	0.106	0.926	0.050	0.855	0.089	0.787	0.076
8	0.765	0.083	0.860	0.065	0.708	0.089	0.796	0.097	0.652	0.112	0.543	0.094
9	0.768	0.082	0.931	0.047	0.827	0.097	0.963	0.036	0.840	0.074	0.785	0.078

Observer	*R*_*x*_				*R*_*y*_				*R*_*z*_			
	Kodak		Planmeca		Kodak		Planmeca		Kodak		Planmeca	
	k*	SE**	k*	SE**	k*	SE**	k*	SE**	k*	SE**	k*	SE**

1	0.833	0.078	0.884	0.079	0.847	0.047	0.901	0.057	0.795	0.083	0.784	0.077
2	0.665	0.101	0.677	0.089	0.792	0.064	0.821	0.072	0.706	0.095	0.615	0.087
3	0.792	0.085	0.894	0.058	0.833	0.079	0.880	0.080	0.957	0.042	0.829	0.048
4	0.790	0.085	0.887	0.088	0.794	0.069	0.828	0.083	0.835	0.076	0.720	0.073
5	0.587	0.106	0.677	0.089	0.697	0.077	0.786	0.065	0.764	0.101	0.615	0.087
6	0.748	0.092	0.894	0.058	0.825	0.059	0.880	0.080	0.915	0.058	0.829	0.048
7	0.833	0.078	0.887	0.078	0.798	0.095	0.871	0.065	0.795	0.083	0.685	0.078
8	0.708	0.097	0.777	0.089	0.802	0.094	0.912	0.088	0.706	0.095	0.615	0.087
9	0.751	0.091	0.758	0.066	0.850	0.081	0.911	0.088	0.915	0.058	0.829	0.048

*Kappa coefficient.

**Standard error.

**Table 6:** Kendall's *W* values for interrater agreement at *t*_2_ and *t*_3_, clustered by software and KPG single component.

	*C*_*x*_		*C*_*y*_		*C*_*z*_	
	Kodak	Planmeca	Kodak	Planmeca	Kodak	Planmeca
	*t*_2_	*t*_2_	*t*_2_	*t*_2_	*t*_2_	*t*_2_
*t*_2_	0.955	0.973	0.942	0.989	0.971	0.966
*t*_3_	0.989	0.999	0.991	0.997	0.997	0.991

	*R*_*x*_		*R*_*y*_		*R*_*z*_	
	Kodak	Planmeca	Kodak	Planmeca	Kodak	Planmeca
	*t*_2_	*t*_2_	*t*_2_	*t*_2_	*t*_2_	*t*_2_

t_2_	0.939	0.991	0.961	0.970	0.975	0.954
t_3_	0.986	0.999	0.988	0.999	0.994	0.992
